# Mesenteric Vessel-Preserving Laparoscopic Surgery for Pediatric Unicentric Castleman Disease in the Transverse Mesocolon

**DOI:** 10.70352/scrj.cr.25-0307

**Published:** 2025-10-01

**Authors:** Takashi Kobayashi, Yoshiaki Kinoshita, Junkichi Takemoto, Yuhki Arai, Yu Sugai, Koichi Saito, Shoichi Takano

**Affiliations:** Department of Pediatric Surgery, Niigata University Graduate School of Medical and Dental Sciences, Niigata, Niigata, Japan

**Keywords:** Castleman disease, unicentric Castleman disease, hyaline vascular type, pediatric, children, mesenteric tumor, laparoscopic surgery, laparoscope, outcome

## Abstract

**INTRODUCTION:**

Castleman’s disease is a rare lymphoproliferative disorder. Unicentric Castleman disease (UCD) is usually benign and can be treated by complete resection. We herein report a pediatric case of UCD that occurred in the transverse mesocolon and was successfully treated with a laparoscopic approach.

**CASE PRESENTATION:**

The patient was a 10-year-old girl at the time of the surgery. She was referred to our hospital with a persistent fever of unknown origin and high C-reactive protein levels. Abdominal enhanced MRI showed a 38 × 25-mm enhanced mass in the left transverse mesocolon, and fluorodeoxyglucose PET-CT showed an abnormal accumulation in the same lesion. UCD was the most suspected diagnosis, and laparoscopic tumor resection was planned and performed. The tumor was adjacent to the inferior mesenteric vein and the left branch of the middle colic artery. There was no evidence of vascular invasion into these vessels. The feeding arteries and drainage veins were securely clipped and cut using an ultrasonic harmonic scalpel. Further dissection revealed swollen bead-like lymph nodes connected to the tumor. After all swollen lymph nodes and the tumor were isolated, they were completely resected en bloc. Colectomy was unnecessary because the main mesenteric vessels were preserved. The histopathological diagnosis was hyaline vascular-type Castleman disease. Her symptoms, such as the fever, disappeared soon after surgery. The patient was discharged on day 9 without any complications. The C-reactive protein level dropped within the normal range 2 weeks after surgery. There was no evidence of recurrence or symptoms 3 years after surgery.

**CONCLUSIONS:**

We encountered a rare case of pediatric UCD. Although only limited cases of the laparoscopic approach for pediatric UCD have been reported, we believe that the laparoscopic approach is useful for UCD in children.

## Abbreviations

CD
: Castleman disease
CRP
: C-reactive protein
ESR
: erythrocyte sedimentation rate
FDG-PET
: fluorodeoxyglucose PET
ICG
: indocyanine green
IL-6
: interleukin-6
IMV
: inferior mesenteric vein
LMCA
: left branch of the middle colic artery
MCD
: multicentric Castleman disease
SAA
: serum amyloid A
sIL-2R
: soluble interleukin-2 receptor
UCD
: unicentric Castleman disease
US
: ultrasound sonography

## INTRODUCTION

CD, a chronic inflammatory disease, is a rare lymphoproliferative disorder. It was first reported as a mediastinal lymphoproliferative disease in 1956.^1)^ CD is rare, with a prevalence rate of 21–25 per million persons per year.^2)^ CD in children accounts for approximately 10% of all CD cases.

The disease is classified into 2 clinical types: UCD and MCD. Two histological subtypes have been defined: hyaline vascular and plasma cell types. The typical clinical symptoms include a fever, general fatigue, and night sweats. Asymptomatic cases have often been observed. Growth retardation or short stature has also been observed in children.^3)^ Laboratory findings showed increased inflammatory responses, such as high CRP levels. Treatment of UCD involves complete removal of the affected lymph nodes alone, and the prognosis is good. The 1st successful case of laparoscopic resection for UCD was reported in 2003.^4)^ In recent years, the usefulness of laparoscopic surgery for adult UCD has been reported^5)^; however, there have been few reports of laparoscopic surgery for UCD in children, and its usefulness is unknown. In addition, intestinal resection is often performed in cases of UCD occurring in the mesenteric lymph nodes.^6)^ However, from the viewpoint of organ preservation, intestinal resection should be avoided, particularly in children.

We herein report a pediatric case of UCD occurring in the transverse mesocolon. The tumor was completely resected laparoscopically, and intestinal resection was avoided by preserving the mesenteric vessels.

## CASE PRESENTATION

A previously healthy 7-year-old girl presented to an outpatient clinic at a local hospital with a fever, headache, and night sweats. Initially, she was diagnosed with pneumonia and treated with antibiotics. Although her symptoms disappeared soon, the elevated CRP level did not improve and continued. Further examinations, including laboratory and imaging studies, were performed; however, the cause of the high CRP level remained unknown. Her symptoms reappeared almost once per month, continued for several days, and then disappeared soon. The patient was observed at the outpatient clinic without any treatment.

At 10 years old, she was admitted to our hospital for a further examination and treatment of these symptoms and her high CRP level. On presentation, her height was 123.2 cm (−2.5 standard deviations [SDs]) and weight was 20.8 kg (−2.0 SDs). She had been born as a healthy full-term, appropriate-for-gestational-age infant. She had no significant medical or family history of hereditary diseases associated with short stature. Her body temperature was 37.1°C during the day and 38.5°C at night. The heart rate was 82 beats per minute, and the blood pressure was 110/68 mmHg. Menarche had not yet occurred. A physical examination revealed a conjunctival pallor. The abdomen was soft and flat, and no abdominal mass was palpable. Superficial lymph nodes were not swollen. Laboratory examinations indicated microcytic hypochromic anemia (hemoglobin: 8.9 g/dL; mean corpuscular volume: 62.3 fl; mean corpuscular hemoglobin: 19.3 pg). The platelet count (562000 µIU/mL) was elevated. The levels of inflammatory markers, including serum CRP (15.3 mg/dL; reference range: <0.1 mg/dL), SAA (895 µg/mL; reference range: <8 µg/mL), and ESR (95 mm/h; reference range: 3–15 mm/h), were elevated. Serum albumin (2.9 g/dL) and serum iron (10 µg/dL; reference range: 40–188 µg/dL) levels were decreased. The sIL-2R level (882 U/mL; reference range: 122–496 U/mL) was slightly increased. Other laboratory data were within the normal range.

**Fig. 1:**
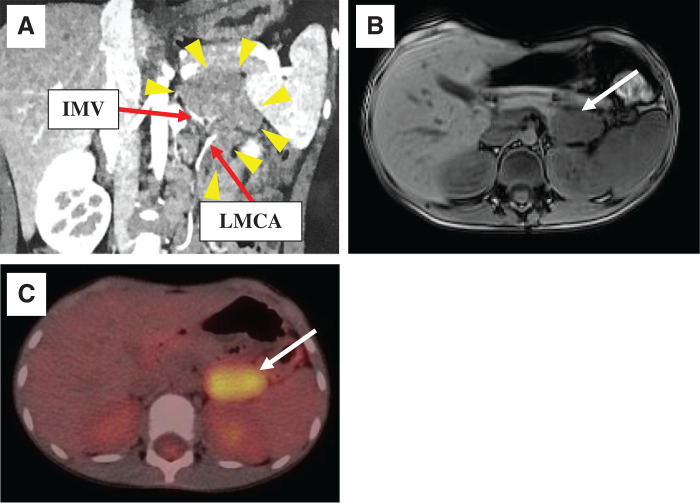
Imaging studies before operation. (**A**) Abdominal enhanced CT showed a 45 × 25-mm clear-boundary, smooth surface, and weakly enhanced tumor (arrowheads). The tumor was adjacent to the IMV and the LMCA without encasement. (**B**) Abdominal MRI showed a 38-mm clear-boundary mass between the distal pancreas and the left kidney, which exhibited uniformly low intensity on T1-weighted imaging (white arrow). (**C**) FDG-PET. An abnormal accumulation of FDG was detected between the distal pancreas and the left kidney (white arrow). FDG-PET, fluorodeoxyglucose PET; IMV, inferior mesenteric vein; LMCA, left branch of the middle colic artery

Abdominal US showed a 30-mm low-echoic, clear-boundary mass lesion. Abdominal enhanced CT showed a 45 × 25-mm weakly enhanced tumor (**Fig. 1A**). The tumor was adjacent to the IMV and the LMCA without encasement (**Fig. 1A**). Abdominal MRI showed a 38-mm clear-boundary mass with uniformly low intensity on T1-weighted imaging (**Fig. 1B**) and slightly high intensity on T2-weighted imaging. The mass also showed diffusion restriction. In addition, several swollen mesenteric lymph nodules were observed in the transverse mesocolon. These lymph nodes were adjacent to the mass and showed the same intensity as that of the mass. To determine the focus of inflammation, a FDG-PET imaging was performed. The abnormal accumulation of FDG was detected between the distal pancreas and the left kidney (**Fig. 1C**). Based on these findings, the tumor was diagnosed as arising from the left transverse mesocolon. The preoperative diagnosis was CD because a 3-year history of symptoms seemed to indicate a benign tumor. The differential diagnosis included malignant lymphoma or inflammatory fibroblastic tumors. We decided to perform laparoscopic surgical tumor resection for both diagnosis and treatment.

### Surgical procedures

**Fig. 2:**
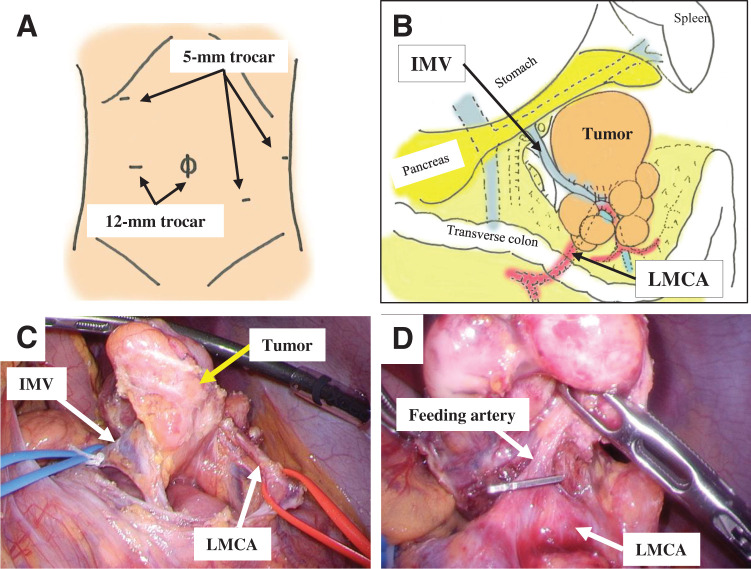
Surgical procedure and findings of the tumor. (**A**) Position of two 12-mm trocars and three 5-mm trocars. (**B**–**D**) The tumor was supplied with arterial flow from the LMCA, and the tumor drainage vein flowed into the IMV. IMV, inferior mesenteric vein; LMCA, left branch of the middle colic artery

Under general anesthesia, a 1.5-cm umbilical incision was made to introduce the 1st trocar for a laparoscope using an open laparotomy technique. After the abdomen was insufflated to 8 mmHg with carbon dioxide, a 2nd trocar (12 mm) was inserted on the left side of the umbilicus. A 10-mm laparoscope (WAIR130A; Olympus, Tokyo, Japan) was placed in the abdomen. Three additional 5-mm trocars were inserted, as shown in **Fig. 2A**. After all trocars had been inserted, the patient was positioned in the head up and right semi-decubitus position. The gastrocolic and splenocolic ligaments were dissected using an ultrasonic scalpel coagulation and cutting device (Harmonic Ace+7; Ethicon Endosurgery, Cincinnati, OH, USA). We mobilized the left transverse colon and exposed the tumor (**Fig. 2B**). The tumor was located on the left side of the transverse mesocolon and received arterial blood supply from the LMCA. The tumor’s drainage vein flowed into the IMV (**Fig. 2C**). The tumor was connected to several swollen lymph nodes in the transverse mesocolon. As UCD is a benign tumor, we attempted to preserve the transverse colon as much as possible. Several tumor-feeding arteries and drainage veins were carefully dissected and isolated. Each vessel was clipped and cut using an ultrasonic scalpel (**Fig. 2D**). Finally, en bloc complete resection and mesenteric vessel preservation were achieved, and colectomy was avoided.

**Fig. 3:**
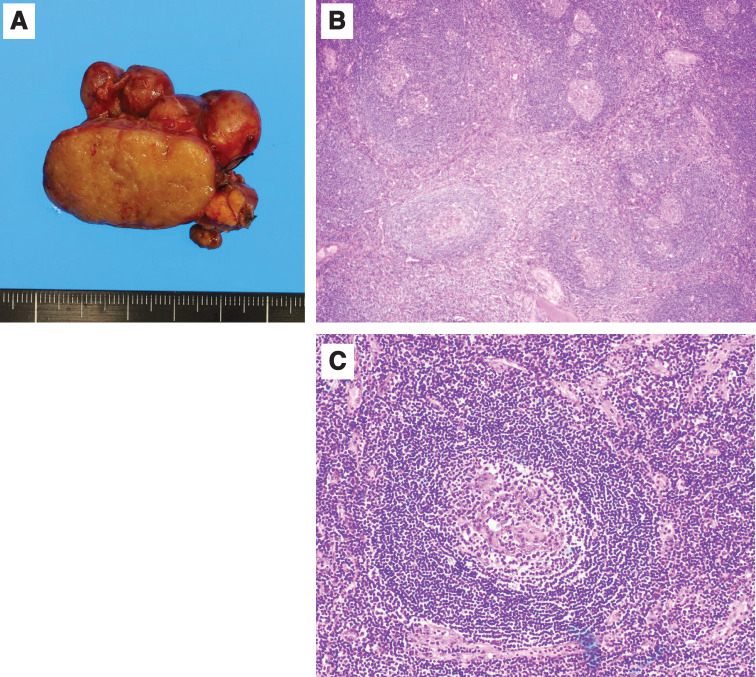
Macroscopic and microscopic findings of the excised specimen. (**A**) The specimen contained enlarged lymph nodes, and the largest lymph node was 39 × 25 mm in size. (**B**) H&E-stained sections at ×40 magnification show multiple enlarged lymphoid follicles. (**C**) H&E-stained sections at ×200 magnification show hyalinized blood vessels in the germinal centers and sclerotic areas. The histopathological diagnosis was hyaline vascular-type Castleman disease. H&E, hematoxylin and eosin

The operative time was 318 min, and blood loss was 5 mL. The excised specimen contained enlarged lymph nodes, and the largest lymph node was 39 × 25 mm in size macroscopically (**Fig. 3A**). Microscopically, multiple enlarged lymphoid follicles (**Fig. 3B**) and hyalinized blood vessels in the germinal centers and sclerotic areas were observed (**Fig. 3C**). The histopathological diagnosis was a hyaline vascular-type CD.

The postoperative course was uneventful, and the patient was discharged on POD 9 without any complications. Her symptoms, such as the fever, headache, and night sweats, disappeared after surgery. The CRP level decreased to the normal range 2 weeks after surgery. There was no evidence of tumor recurrence 3 years and 1 month after surgery. The patient’s height was 141.5 cm (−2.5 SD), and her body weight was 39.1 kg (−1.3 SD) 3 years and 1 month after surgery.

## DISCUSSION

Complete laparoscopic tumor resection was successfully performed in a pediatric patient with UCD of the transverse mesocolon who presented with a fever, night sweats, and growth retardation. The laparoscope’s magnification effect facilitated preservation of the mesenteric vessels, which avoided the need for combined colectomy, and allowed accurate identification of the tumor vessels, thereby reducing bleeding. This is the first report of laparoscopic surgery for mesenteric UCD in a child.

CD is a rare lymphoproliferative disorder characterized by involvement of lymph nodes in the body. CD can be divided into 2 categories: UCD and MCD. UCD involves only 1 lymph node region and is usually asymptomatic, whereas MCD is associated with systemic symptoms and involvement of multiple lymph nodes. UCD usually behaves as a benign tumor; in contrast, MCD is potentially life-threatening. UCD can be further divided into 2 subtypes based on the histological features: the hyaline vascular type and the plasmacytic type, with the hyaline vascular type being the majority (74.4%–91.4%).^7,8)^

There are some differences in CD between children and adults. Regarding the location of tumors, the chest accounts for most cases in adults (75%).^9)^ Among pediatric cases, more than half (53.5%) had head and neck manifestations, 24.4% were abdominal, and 16.3% were thoracic.^10)^ The proportion of the abdomen was relatively higher in children than in adults with CD. CD often presents with vague symptoms, such as fever, general fatigue, slight body weight loss, and night sweats. Growth retardation is a characteristic feature in children. Approximately 50% of pediatric UCD cases are asymptomatic. Blood examinations showed an increase in inflammatory responses, such as elevation of ESR, SAA, and CRP levels. Other findings include anemia and an increased platelet count. Serological tests revealed elevated immunoglobulin G and IL-6, while tumor markers showed elevated sIL-2R levels. However, 50% of the UCD cases showed no abnormalities on blood examination. Imaging findings in CD are nonspecific, and a diagnosis based on these findings is challenging. US shows a round, homogeneous, hypoechoic area with well-defined, smooth edges. Color Doppler US shows increased blood flow in and around the lesional lymph node. Plain CT indicates isolated, well-demarcated, round or lobulated mass lesions with a density equivalent to that of normal muscle.^11)^ Enhanced CT shows soft tissue attenuation with enhancement. MRI is isointense to slightly hypointense on T1-weighted imaging and hyperintense on T2-weighted imaging. Small satellite mesenteric lymph nodes and mass effects on surrounding structures may be detected.^12,13)^ FDG-PET/CT shows metabolic activity in the affected lymph nodes.^14)^ High specific uptake values should raise suspicion of lymphoma, which is an important differential diagnosis. Thus, because it is difficult to diagnose CD using imaging studies alone, a histopathological diagnosis is necessary.

Complete surgical resection is effective and is recommended for patients with UCD. In recent years, cases of laparoscopic resection have been reported in adults.^15)^ Compared with open surgery, laparoscopic surgery is less invasive and provides a magnified visual effect, making it a good option for UCD. However, only a few laparoscopic approaches have been reported in pediatric cases.

A PubMed search was conducted using the keywords “Castleman disease” and “laparoscope,” and case reports of patients aged ≤15 years, excluding articles not published in English, are summarized in **Table 1**. To date, including the present case, there have been 3 cases of laparoscopic resection in children aged ≤15 years, of which 2 were retroperitoneal.^16,17)^ All 3 pediatric cases underwent complete tumor resection laparoscopically. No surgical complications or tumor recurrences were reported during follow-up. The 1st successful laparoscopic resection of mesenteric UCD was reported in 2011.^18)^ It was performed on a 60 × 50 × 35-mm tumor in the mesorectum, and complete resection was achieved. Since then, other laparoscopic resections for mesenteric UCD have been reported.^6,19)^ In 1 case of sigmoid mesenteric UCD, laparoscopic high anterior resection was performed.^20)^ It is often technically difficult to isolate only tumors from the mesentery because they are connected to several small feeding vessels and drainage veins. Therefore, colectomy is unavoidable in most cases of mesenteric CD. However, colectomy should be avoided, particularly in children. Bracale et al. first reported laparoscopic tumor resection without colectomy for mesenteric UCD of the transverse mesocolon. They concluded that it is important to maintain an appropriate dissection plane.^6)^ In the present case, the laparoscopic magnified view enabled the accurate detection of small feeding vessels. Taping the mesenteric vessels is very useful for maintaining the dissection plane and properly mobilizing the tumor without both tumor and vessel injury. As a result, we were able to preserve the mesenteric vessels and resect the tumor without colectomy. Surgeons must pay attention to preventing bleeding because most cases of UCD are hypervascular tumors. There have been several reports of conversion to open surgery due to bleeding in patients with UCD.^15,21)^ There has also been a retroperitoneal UCD case of ureteral injury that occurred during laparoscopic surgery, leading to conversion to open surgery.^21)^

**Table 1:** A literature review of pediatric (<16 years old) case reports of laparoscopic surgery for unicentric Castleman disease

	Author	Publication year	Age at surgery (years)	Sex	Tumor location	Symptoms	Imaging study	Abnormal laboratory data	Preoperative diagnosis	Surgical technique	Treatment outcome	Excised tumor size (mm)	Histological type	Surgical complications	Postoperative hospital stay (days)	Follow-up (months)	Outcomes
1	Modi et al.^16)^	2008	15	M	Anterior and medial to the left kidney	Hypertension	US, CT, MRI	None	N/A	LR	CR	67 × 54	HV type	None	2	3	NR
2	Orzel et al.^17)^	2024	11	F	Right retroperitoneal space	Right abdominal pain, nausea, emesis, hematuria	CT, MRI	Urinalysis	PRS, NF, GCT, schwannoma, PC, lymphoma	LR	CR	26 × 26 × 39	HV type	None	N/A	N/A	N/A
3	Present study	2025	10	F	Left transverse mesocolon	Fever, headache, night sweats	US, CT, MRI, PET-CT	Hb, Plt, Alb, CRP, SAA, ESR, etc.	Castleman disease	LR	CR	39 × 25	HV type	None	9	38	NR

Alb, albumin; CR, complete resection; CRP, C-reactive protein; ESR, erythrocyte sedimentation rate; F, female; GCT, germ cell tumor; Hb, hemoglobin; HV, hyaline vascular; LR, laparoscopic resection; M, male; N/A, not available; NF, neurofibroma; NR, no recurrence; PC, pheochromocytoma; Plt, platelet; PRS, primary retroperitoneal sarcoma; SAA, serum amyloid A; US, ultrasound

Patients with UCD who underwent surgical resection had a favorable overall survival rate of 95.3%, a 3-year disease-free survival of 89.7%, and a 5-year disease-free survival of 81.2%.^22)^ After complete tumor removal, symptoms such as fever soon disappeared in our patient, and inflammatory responses, such as the CRP level, improved and normalized. Regarding short stature in children, a review of 7 pediatric CD cases with short stature reported that catch-up in height occurred in 5 cases during the follow-up period.^3)^ There have been reports of local recurrence 14 years after complete resection, and long-term follow-up is necessary, even in cases of complete resection.^23)^

When performing laparoscopic resection, ICG administration may be useful for detecting the feeding artery. Unfortunately, ICG was not used in this case because it was not available. There have been reports that ICG is useful for identifying tumor feeding arteries.^24)^ We are considering using ICG in a planned manner in the future.

## CONCLUSIONS

We report a rare pediatric case of mesenteric UCD that was treated laparoscopically with complete surgical resection. Although only limited cases of the laparoscopic approach for pediatric UCD have been reported, we believe that the laparoscopic approach is useful for treating UCD in children.

## DECLARATIONS

### Availability of data and materials

The data that support the findings of this study are available from the corresponding author upon reasonable request.
